# Roles of Lung Ultrasound Score in the Extubation Failure From Mechanical Ventilation Among Premature Infants With Neonatal Respiratory Distress Syndrome

**DOI:** 10.3389/fped.2021.709160

**Published:** 2021-12-06

**Authors:** Zhenyu Liang, Qiong Meng, Chuming You, Bijun Wu, Xia Li, Qianmei Wu

**Affiliations:** ^1^Department of Neonatology, Guangdong Second Provincial General Hospital, Guangzhou, China; ^2^Ultrasonic Department, Guangdong Second Provincial General Hospital, Guangzhou, China

**Keywords:** lung ultrasound score, premature infants, neonatal respiratory distress syndrome, mechanical ventilation, pulmonary

## Abstract

**Objective:** To investigate the predictive value of lung ultrasound score (LUS) in the extubation failure from mechanical ventilation (MV) among premature infants with neonatal respiratory distress syndrome (RDS).

**Methods:** The retrospective cohort study was conducted with a total of 314 RDS newborns who received MV support for over 24 h. After extubation from MV, infants were divided into extubation success and extubation failure groups. Extubation failure was defined as re-intubation within 48 h after extubation. Univariate and multivariate logistic regression analyses were used to identify the predictors of the extubation failure. The predictive effectiveness of the combined model and LUS in the extubation failure was assessed by receiver operating characteristic curve, area under curve (AUC), and internal validation.

**Results:** 106 infants failed extubation from MV. The combined model for predicting the extubation failure was performed according to the predictors of gestational age, body length, birth weight, and LUS. The AUC of this combined model was 0.871 (sensitivity: 86.67%, specificity: 74.31%). The AUC of LUS was 0.858 (sensitivity: 84.00%, specificity: 80.69%), and the cutoff value was 18. There was no statistical difference in the predictive power between the combined model and LUS (*Z* = 0.880, *P* = 0.379). The internal validation result showed that the AUC of LUS was 0.855.

**Conclusions:** LUS presented a good ability in predicting the extubation failure among RDS newborns after MV.

## Introduction

Neonatal respiratory distress syndrome (RDS), a common respiratory disease, is a leading cause of early morbidity and mortality among infants and children (1, 2). It is characterized by diffuse lesions of the pulmonary capillaries and increased permeability (3). Pieces of evidence showed that premature deaths caused by neonatal RDS accounted for 50–70% of all premature deaths, and survivors seemed more likely to suffer from severe sequelae (4, 5). Mechanical ventilation (MV) is frequently applied in a neonatal intensive care unit (6) and can efficiently relieve the clinical symptoms of premature infants undergoing severe RDS (7), whereas long-term MV may be associated with the risk of ventilator-relevant lung injury, bronchopulmonary dysplasia, and infection (8, 9). Of note, the ultimate aim of treatment is to help patients weaning from MV support, not to provide MV support (9).

The extubation success of MV has been gradually attracted attention in the treatment of respiratory support in recent years. Studies reported that the extubation of MV is associated with chronic obstructive airway disease, heart failure, decompensation of cardiopulmonary function, positive fluid balance, pneumonia, and diaphragmatic dysfunction (10, 11). A common reason for the weaning failure is the imbalance between ventilation demand and the capacity of spontaneous breathing, such as respiratory pump failure (12). To the best of our knowledge, pulmonary function is an important factor affecting the extubation success from MV in RDS infants. At present, lung ultrasound score (LUS), a reliable measuring method for the bedside evaluation of pulmonary ventilation, is widely used for critically ill patients, especially for neonatal RDS (13, 14). Early researches mentioned that LUS could predict the use of surfactants under continuous positive airway pressure in premature babies (15, 16). Furthermore, LUS and the area of lung consolidation are closely related to the severity of neonatal RDS, which has also been confirmed in several studies (17). However, the application of LUS in the extubation of MV in babies with RDS has not yet been concluded, and further researches are needed to explore.

Herein, we observed the pulmonary ultrasound performance of neonatal RDS and measured the LUS in premature infants, thereby further assessing the reliability and accuracy of LUS in predicting the extubation failure, which may provide guidance for the extubation of MV.

## Methods

### Patients

This was a retrospective cohort study. A total of 314 eligible infants who received MV support within 72 h after birth were enrolled consecutively between January 1, 2019, and June 30, 2020. After extubation from MV, infants were divided into extubation success and extubation failure groups. This study was approved by the Institutional Review Board of Guangdong Second Provincial General Hospital (approval number: No.20191101-01-YXKXYJ-SYX), and the written informed consent was obtained from the parents or guardians of the minors.

### Eligibility

Inclusion criteria were (1) gestational age <37 weeks, (2) infants with RDS, and (3) MV support for ≥24 h.

Exclusion criteria were (1) congenital diseases, such as congenital heart disease, respiratory malformation, and chromosome abnormality; (2) a history of pneumothorax, air leak, or meconium aspiration syndrome; (3) arrhythmias and hemodynamic instability; (4) >stage III intracranial hemorrhage; (5) taking other experimental drugs or participating in other clinical trials within 1 month before the inclusion in this study; (6) respiratory distress caused by other diseases; and (7) incomplete clinical data.

The definition of neonatal RDS based on the *Practice of Neonatology* (5th edition) (3) were as follows: (1) infants with progressive dyspnea within 6 h after birth accompanied by cyanosis, expiratory groan or inspiratory three concave sign; (2) cases with decreased transparency of bilateral lungs, air bronchogram, indistinct heart, and septal margins, or white lung based on chest X-rays.

### Clinical Data

All eligible infants were examined by chest radiograph, lung ultrasound, cardiac color ultrasound, and blood gas analysis within 1 h before the extubation.

#### General Data

The characteristics of RDS infants were recorded at admission, including gestational age (weeks), sex, nationality, weight (kilogram), and body length (centimeter).

#### Echocardiographic Indicators

The echocardiographic indicators were recorded including left ventricular end-diastolic diameter (LVEDD), left ventricular end-systolic diameter, aortic dimension, pulmonary valve, aortic valve, tricuspid valve, bicuspid valve, descending aorta, and left ventricular ejection fraction.

#### Lung Ultrasound Examination

**Figure 1:**
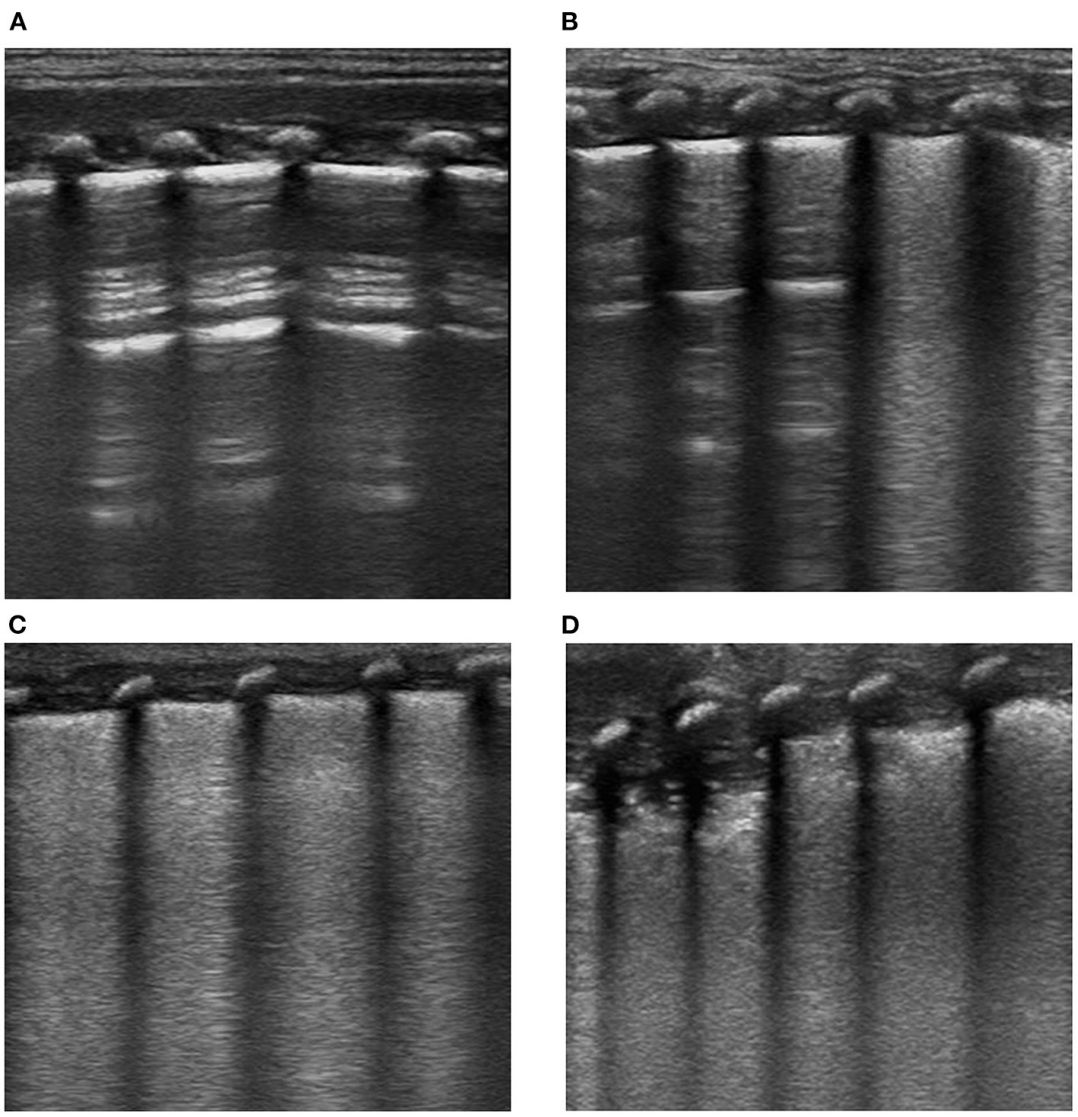
Imaging examination of lung ultrasound in newborns with RDS. **(A)** Score = 0, **(B)** score = 1, **(C)** score = 2, and **(D)** score = 3.

The lung ultrasound examination was performed using the Doppler ultrasound diagnostic instrument (Philips CX50), with a probe frequency of 8–12 MHz. The 12-region method was conducted to examine the anterior, lateral, and posterior walls on both sides of the lung (18). The scoring standard of lung ultrasound is as follows: (1) normal aeration (score = 0); (2) moderate loss of aeration (interstitial syndrome, defined by multiple spaced B lines, or localized pulmonary edema, defined by coalescent B lines in <50% of the intercostal space examined in the transversal plane, or subpleural consolidations) (score = 1); (3) severe loss of aeration (alveolar edema, defined by diffused coalescent B lines occupying the whole intercostal space) (score = 2); and (4) complete loss of lung aeration (lung consolidation defined as a tissue pattern with or without air bronchogram) (score = 3). The images of lung ultrasound examination are shown in Figure 1. LUS is calculated as the sum of the 12 regional scores, ranging from 0 to 36; LUS = 0 is normal, and LUS > 0 is abnormal. The observation point was before the extubation of MV. The lung ultrasound was conducted by pediatricians who had participated in the training courses of neonatal pulmonary ultrasound in China. The images stored by two pediatricians were reviewed and controlled for quality by a sonographer (chief physician) with proficient skills in pulmonary ultrasound.

#### Arterial Blood Gas Analysis

The parameters were analyzed, including oxygenation index (OI), arterial partial pressure of carbon dioxide (PaCO_2_), and rapid shallow breathing index (RSBI). OI is the ratio of arterial partial pressure of oxygen and fraction of inspired oxygen, which can reflect the severity of acute diseases. RSBI is a ratio of respiratory rate (beats/min) and tidal volume (liter).

#### Extubation From Mechanical Ventilation

The extubation of ventilators was based on: (1) when fraction of inspired oxygen ≤0.4, peak inspiratory pressure = 10–15 cm H_2_O, positive end-expiratory pressure) <2–4 cm H_2_O, and frequency ≤ 10 beats/min; (2) normal arterial blood gas; (3) acid-base electrolyte balance; (4) recovery of spontaneous breathing and sufficient effective ventilation; (5) control or improvement of the primary disease; and (6) reduced secretion and good tolerance of sputum suction. After the extubation, respiratory insufficiency occurred in the infants, which could not be relieved after treatments with oxygen inhalation, nebulization inhaled corticosteroids, or β-receptor agonists. If babies were re-ventilated with endotracheal intubation within 48 h after extubation, the extubation was deemed as failure; otherwise, it was successful (19).

### Statistical Analysis

Statistical analysis was performed using SAS 9.4 (SAS Institute Inc.). The normality of data was examined by the Shapiro–Wilk test (W test). Measurement data with normal distribution were presented as mean ± standard deviation ($\bar{x}\pm s$) using *t*-test and, with skewed distribution, were presented as median and quartile [M(Q_25_, Q_75_)] by Mann–Whitney *U*-test. Enumeration data were presented as n (%) with chi-squared [*χ*^2^ (total n ≥40 and all T ≥5)] or Fisher (total *n* < 40 and all T < 5) tests. Ranked data were presented as n (%) utilizing the Mann–Whitney U test. *P* < 0.05 was considered statistically significant.

All eligible newborns in this study were randomly divided into the training group (*n* = 220) and testing group (*n* = 94) with a ratio of 7:3. A prediction model was established using the data of the training group. Univariate and multivariable logistic regression analyses were used to identify the predictors of the extubation failure from MV in RDS newborns. A combined model for predicting the extubation failure was established based on the predictors. Then, the predictive power between the combined model and LUS was compared. The predictive performance of LUS for the extubation failure was evaluated using the testing group (as the internal validation). Variance inflation factor (VIF) was used to assess the collinearity of independent variables. The power analysis was performed using SPSS 15.0 (SPSS, Inc., Chicago, IL). The power of the AUC of the combined model was 1.00, and the power of the AUC of LUS was 1.00.

## Results

### Baseline Data of Premature Infants With Neonatal Respiratory Distress Syndrome

**Figure 2:**
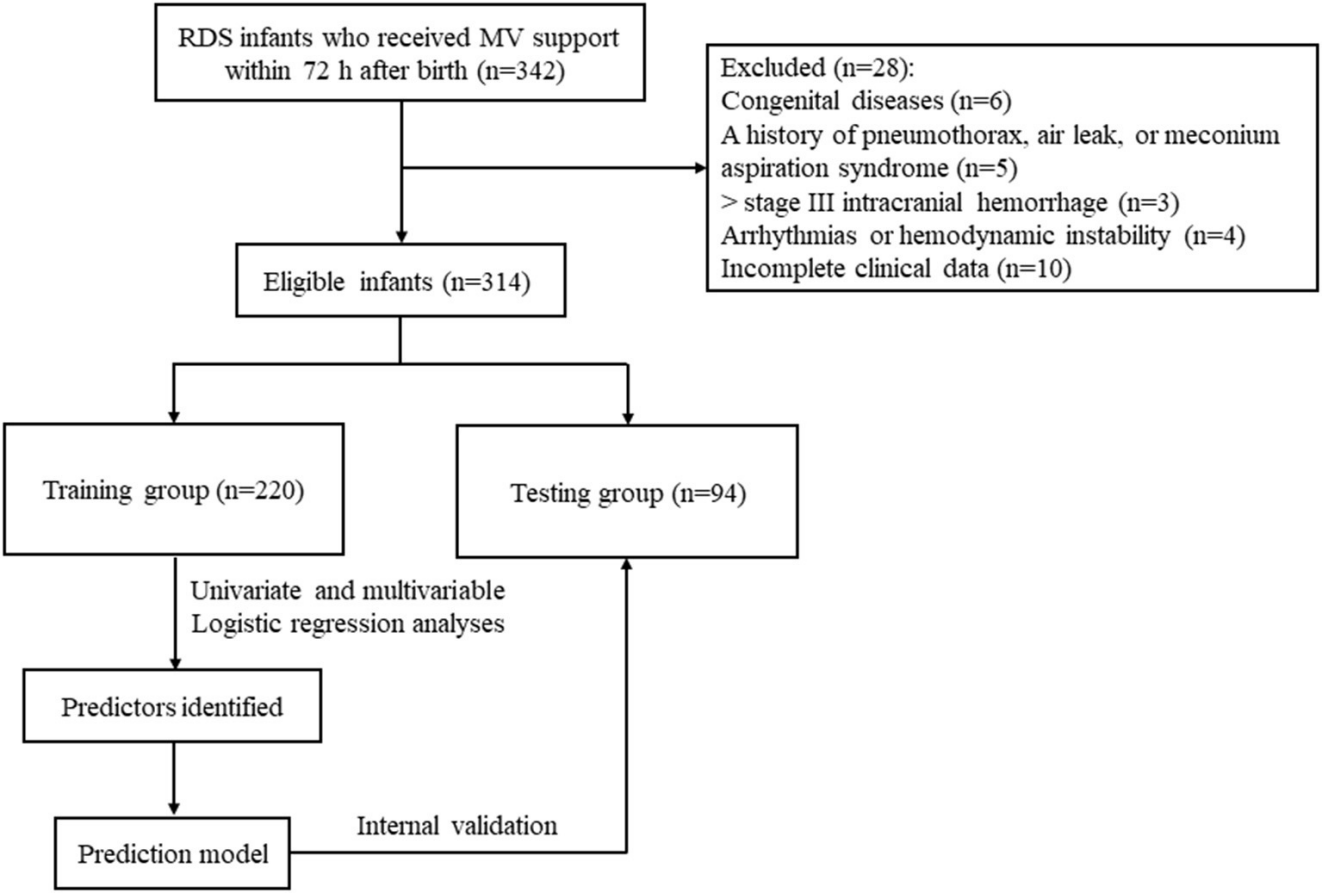
Flow chart of included infants with RDS.

In the current study, a total of 342 premature infants with neonatal RDS who received MV for ≥24 h in the intensive care unit were enrolled in this study. After excluding newborns with congenital diseases (*n* = 6), a history of pneumothorax, air leak, or meconium aspiration syndrome (*n* = 5), > stage III intracranial hemorrhage (*n* = 3), arrhythmias or hemodynamic instability (*n* = 4), and incomplete clinical data (*n* = 10), 314 were finally included. The flow chart of the included patients has been added in our manuscript; please see revised Figure 2. One hundred six cases failed extubation from MV. Of the total, 66.88% were males (*n* = 210), and 33.12% (*n* = 104) were females, with the mean gestational age of (32.94 ± 3.44) weeks, the median birth weight of 1.26 (1.01, 1.80) kg, and the mean body length of (40.02 ± 5.38) cm.

### Difference Analysis Between the Training and Testing Groups

Of the total 314 newborns, 220 were classified into the training group, and 94 were in the testing group. There were no statistical differences in all included variables regarding gestational age, sex, nationality, body length, birth weight, breathing, OI, arterial partial pressure of oxygen, PaCO_2_, RSBI, duration of MV, LVEDD, left ventricular end-systolic diameter, aortic dimension, pulmonary valve, tricuspid valve, bicuspid valve, descending aorta, left ventricular ejection fraction, pulmonary artery systolic pressure, LUS, patent foramen ovale, and artery bypass from left to right horizontally, with all *P* > 0.05 (Table 1).

**Table 1:** Difference analysis between the training and testing groups.

**Variables**	**Testing group** **(***n*** = 94)**	**Training group** **(***n*** = 220)**	**Statistics**	***P***
Gestational age, weeks, $\bar{x}\pm s$	32.95 ± 3.32	32.93 ± 3.50	*t* = 0.03	0.972
Sex, *n* (%), $\bar{x}\pm s$			*χ^2^* = 1.807	0.179
Male	68 (72.34)	142 (64.55)		
Female	26 (27.66)	78 (35.45)		
Nationalities, *n* (%)			–	1.000
Han	94 (100.00)	218 (99.09)		
Uyghur	0 (0.00)	2 (0.91)		
Body length, cm, $\bar{x}\pm s$	39.56 ± 5.35	40.22 ± 5.39	*t* = −0.99	0.322
Birth weight, kg, M(Q_25_, Q_75_)	1.25 (1.04, 1.72)	1.27 (1.01, 1.80)	*Z* = −0.219	0.826
Breathing, beats/min, $\bar{x}\pm s$	49.15 ± 6.79	50.15 ± 5.30	*t* = −1.28	0.203
OI, $\bar{x}\pm s$	4.20 (2.75, 5.96)	3.74 (2.82, 5.15)	*Z* = 0.704	0.482
PaO_2_, mmHg, $\bar{x}\pm s$	78.21 ± 23.07	79.57 ± 20.13	*t* = −0.52	0.601
PaCO_2_, mmHg, $\bar{x}\pm s$	41.98 ± 10.31	40.80 ± 9.25	*t* = 1.00	0.320
RSBI, $\bar{x}\pm s$	87.87 ± 5.24	88.13 ± 5.14	*t* = −0.41	0.682
Duration of MV, h, M(Q_25_, Q_75_)	102.00 (61.00, 176.00)	128.00 (79.00, 200.00)	*Z* = −1.330	0.184
LVEDD, $\bar{x}\pm s$	13.06 ± 2.27	12.80 ± 2.73	*t* = 0.87	0.385
LVESD, $\bar{x}\pm s$	8.56 ± 1.54	8.24 ± 1.79	*t* = 1.52	0.130
Aortic dimension, $\bar{x}\pm s$	6.47 ± 0.75	6.51 ± 1.08	*t* = −0.42	0.676
Pulmonary valve, $\bar{x}\pm s$	0.80 (0.70, 1.00)	0.80 (0.70, 1.00)	*Z* = −1.054	0.292
Aortic valve, $\bar{x}\pm s$	0.92 ± 0.22	0.91 ± 0.21	*t* = 0.27	0.784
Tricuspid valve, $\bar{x}\pm s$	0.61 ± 0.20	0.64 ± 0.14	*t* = −1.06	0.293
Bicuspid valve, $\bar{x}\pm s$	0.71 ± 0.17	0.71 ± 0.17	*t* = −0.11	0.912
Descending aorta, $\bar{x}\pm s$	1.09 ± 0.23	1.09 ± 0.21	*t* = −0.30	0.762
LVEF, $\bar{x}\pm s$	67.03 ± 4.58	67.15 ± 4.20	*t* = −0.22	0.823
PASP, $\bar{x}\pm s$	38.40 ± 9.78	38.69 ± 12.32	*t* = −0.19	0.850
LUS, $\bar{x}\pm s$	18.78 ± 3.44	19.62 ± 4.08	*t* = −1.76	0.079
PDA, *n* (%)			*χ^2^* = 0.268	0.604
No	50 (53.19)	110 (50.00)		
Yes	44 (46.81)	110 (50.00)		
PFO, *n* (%)			–	1.000
No	3 (3.19)	7 (3.18)		
Yes	91 (96.81)	213 (96.82)		
Artery bypass from left to right horizontally, *n* (%)			*χ^2^* = 3.192	0.074
No	69 (73.40)	181 (82.27)		
Yes	25 (26.60)	39 (17.73)		

*–, Using Fisher test*.

*OI, oxygenation index; RSBI, rapid shallow breathing index; PaO_2_, arterial partial pressure of oxygen; PaCO_2_, arterial partial pressure of carbon dioxide; MV, mechanical ventilation; LVEDD, left ventricular end-diastolic dimension; LVESD, left ventricular end-systolic diameter; LVEF, left ventricular ejection fraction; PASP, pulmonary artery systolic pressure; LUS, lung ultrasound score; PDA, patent ductus arteriosus; PFO, patent foramen ovale*.

### Univariate Logistic Regression Analysis for the Extubation Failure

The results of the unavailable analysis in the training set are shown in Table 2. Gestational age (31.65 *vs*. 33.60, *t* = 4.04), body length (36.27 *vs*. 42.26, *t* = 9.84), and birth weight (0.99 *vs*. 1.58, *Z* = −8.699) in the extubation failure group were lower than those in the extubation success group, with all *P* < 0.001. PaCO_2_ (43.04 *vs*. 39.64, *t* = 2.61), LVEDD (13.32 *vs*. 12.53, *t* = 2.30), aortic aorta (0.95 *vs*. 0.89, *t* = 2.19), and LUS (20.72 *vs*. 19.06, *t* = −2.92) in the extubation failure group were higher in comparison with those in the extubation success group, with all *P* < 0.05.

**Table 2:** Univariate logistic regression analysis for the extubation failure.

**Variables**	**Extubation success** **(***n*** = 145)**	**Extubation failure** **(***n*** = 75)**	**Statistics**	***P***
Gestational age, weeks, $\bar{x}\pm s$	33.60 ± 3.57	31.65 ± 2.99	*t* = 4.04	<0.001
Gender, *n* (%), $\bar{x}\pm s$			*χ^2^* = 0.224	0.636
Male	92 (63.45)	50 (66.67)		
Female	53 (36.55)	25 (33.33)		
Nationalities, *n* (%)			–	0.115
Han	145 (100.00)	73 (97.33)		
Uyghur	0 (0.00)	2 (2.67)		
Body length, cm, $\bar{x}\pm s$	42.26 ± 4.91	36.27 ± 3.91	*t* = 9.84	<0.001
Birth weight, kg, M(Q_25_, Q_75_)	1.58 (1.24, 2.00)	0.99 (0.92, 1.19)	*Z* = −8.699	<0.001
Breathing, beats/min, $\bar{x}\pm s$	49.94 ± 5.27	50.57 ± 5.38	*t* = −0.84	0.401
OI, $\bar{x}\pm s$	3.76 (2.81, 4.90)	3.65 (2.91, 5.57)	*Z* = 0.782	0.434
PaO_2_, mmHg, $\bar{x}\pm s$	80.57 ± 19.89	77.64 ± 20.59	*t* = 1.02	0.307
PaCO_2_, mmHg, $\bar{x}\pm s$	39.64 ± 9.01	43.04 ± 9.38	*t* = −2.61	0.010
RSBI, $\bar{x}\pm s$	87.92 ± 5.27	88.54 ± 4.87	*t* = −0.85	0.397
Duration of MV, h, M(Q_25_, Q_75_)	122.0 (66.0, 183.0)	165.0 (81.0, 255.0)	*Z* = 1.865	0.062
LVEDD, $\bar{x}\pm s$	12.53 ± 3.00	13.32 ± 2.02	*t* = −2.30	0.023
LVESD, $\bar{x}\pm s$	8.09 ± 1.97	8.51 ± 1.37	*t* = −1.84	0.067
Aortic dimension, $\bar{x}\pm s$	6.45 ± 1.17	6.63 ± 0.89	*t* = −1.23	0.220
Pulmonary valve, $\bar{x}\pm s$	0.80 (0.70, 1.00)	0.80 (0.80, 1.00)	*Z* = 1.527	0.127
Aortic valve, $\bar{x}\pm s$	0.89 ± 0.23	0.95 ± 0.15	*t* = −2.19	0.030
Tricuspid valve, $\bar{x}\pm s$	0.63 ± 0.14	0.65 ± 0.14	*t* = −0.88	0.377
Bicuspid valve, $\bar{x}\pm s$	0.71 ± 0.19	0.72 ± 0.15	*t* = −0.54	0.593
Descending aorta, $\bar{x}\pm s$	1.08 ± 0.23	1.12 ± 0.17	*t* = −1.54	0.126
LVEF, $\bar{x}\pm s$	67.04 ± 4.32	67.36 ± 3.97	*t* = −0.54	0.593
PASP, $\bar{x}\pm s$	37.0 (35.0, 45.0)	36.0 (35.0, 41.0)	*Z* = −1.384	0.166
LUS, $\bar{x}\pm s$	19.06 ± 3.94	20.72 ± 4.16	*t* = −2.92	0.004
PDA, *n* (%)			*χ^2^* = 0.506	0.477
No	75 (51.72)	35 (46.67)		
Yes	70 (48.28)	40 (53.33)		
PFO, *n* (%)			–	0.427
No	6 (4.14)	1 (1.33)		
Yes	139 (95.86)	74 (98.67)		
Artery bypass from left to right horizontally, *n* (%)			*χ^2^* = 0.233	0.629
No	118 (81.38)	63 (84.00)		
Yes	27 (18.62)	12 (16.00)		

*–, Using Fisher test*.

*OI, oxygenation index; RSBI, rapid shallow breathing index; PaO_2_, arterial partial pressure of oxygen; PaCO_2_, arterial partial pressure of carbon dioxide; MV, mechanical ventilation; LVEDD, left ventricular end-diastolic dimension; LVESD, left ventricular end-systolic diameter; LVEF, left ventricular ejection fraction; PASP, pulmonary artery systolic pressure; LUS, lung ultrasound score; PDA, patent ductus arteriosus; PFO, patent foramen ovale*.

### Collinearity Analysis of Independent Variables

The collinear assessment of independent variables is shown in Table 3. The VIF values of gestational age, body length, birth weight, PaCO_2_, LVEDD, and LUS were 2.020, 3.129, 3.676, 1.025, 1.082, and 1.036, respectively. All VIF values were ≤ 10, and all tolerance values were <1, indicating no collinearity among the independent variables.

**Table 3:** Collinear assessment of independent variables.

**Variables**	**VIF**	**Tolerance**
Gestational age	2.020	0.495
Body length	3.129	0.320
Birth weight	3.676	0.272
PaCO_2_	1.025	0.976
LVEDD	1.082	0.924
LUS	1.036	0.956

*VIF, variance inflation factor; PaCO_2_, arterial partial pressure of carbon dioxide; LVEDD, left ventricular end-diastolic diameter; LUS, lung ultrasound score*.

### Multivariate Logistic Regression Analysis for the Extubation Failure

The stepwise logistic regression was used to evaluate the predictive factors of the extubation failure among newborns with RDS (Table 4). The findings showed that gestational age, body length, birth weight, and LUS were predictive factors of extubation failure. The risk of the extubation failure decreased by 0.149 [95% confidence interval (CI): 0.967–0.990, *P* = 0.037] and 0.181 times (95% CI: 0.705–0.950, *P* = 0.009) for every 1-week increase in gestational age and every 1-cm increase in body length, respectively. When the birth weight gained 1 kg each time, the extubation failure risk reduced by 0.905-folds (95% CI: 0.018–0.504, *P* = 0.006). In addition, a 0.116–fold (95% CI: 1.012–1.231, *P* = 0.028) increase was exhibited in the risk of the extubation failure with per 1 unit increase in LUS (details in Table 4).

**Table 4:** Multivariate Logistic regression analysis for the extubation failure.

**Variables**	***β***	***S. E***	**Wald**	***P***	***OR***	**95% CI**	
						**Lower**	**Upper**
Gestational age	−0.161	0.077	4.344	0.037	0.851	0.967	0.990
Body length	−0.200	0.076	6.902	0.009	0.819	0.705	0.950
Birth weigh	−2.351	0.849	7.658	0.006	0.095	0.018	0.504
LUS	0.110	0.050	4.811	0.028	1.116	1.012	1.231

*OR, odds ratio; CI, confidence interval; LUS, lung ultrasound score*.

### Prediction for the Extubation Failure From Mechanical Ventilation in Newborns With Respiratory Distress Syndrome

**Figure 3:**
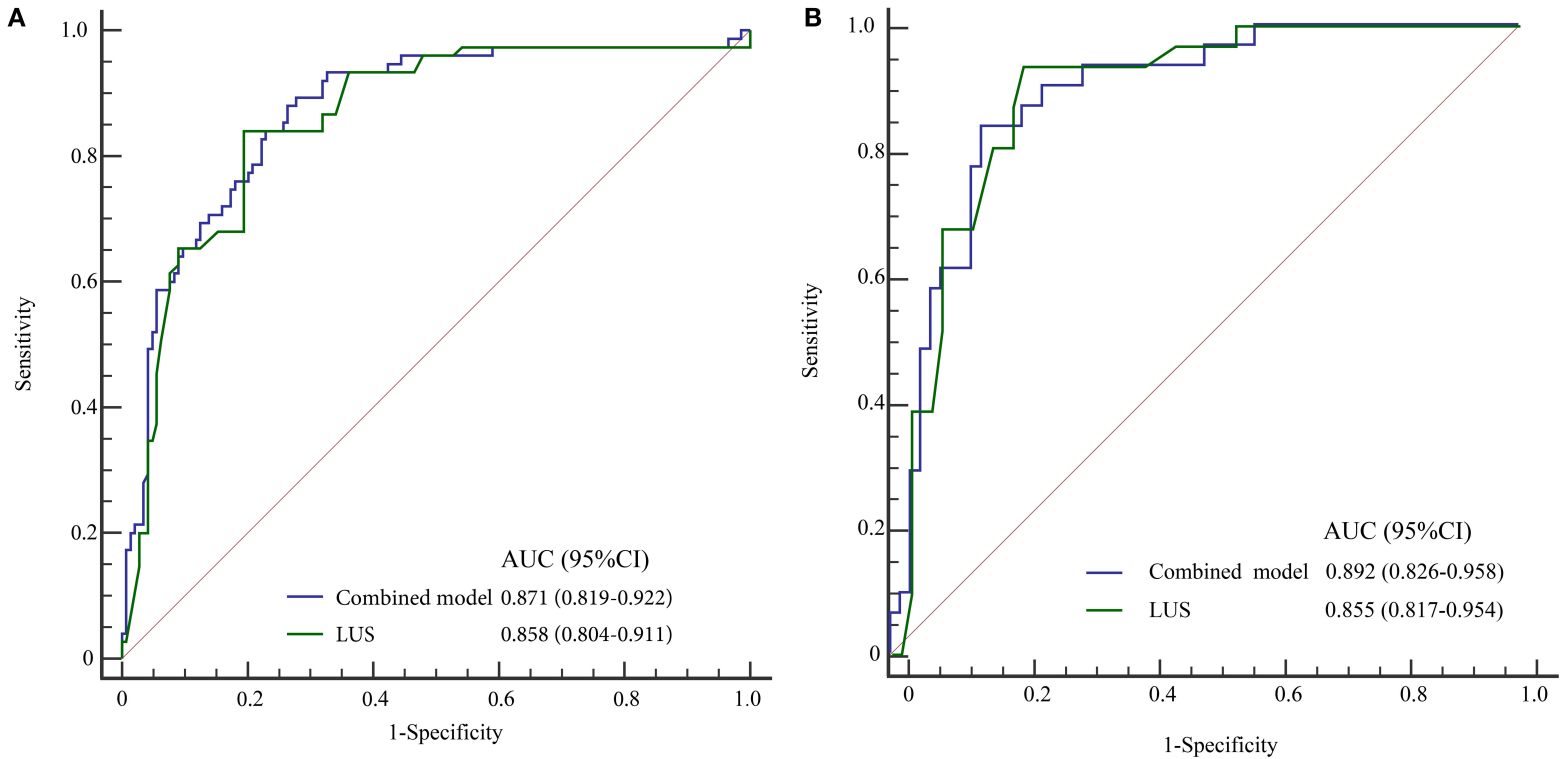
ROC curves for predicting extubation failure of MV. **(A)** Training group, **(B)** testing group.

**Figure 4:**
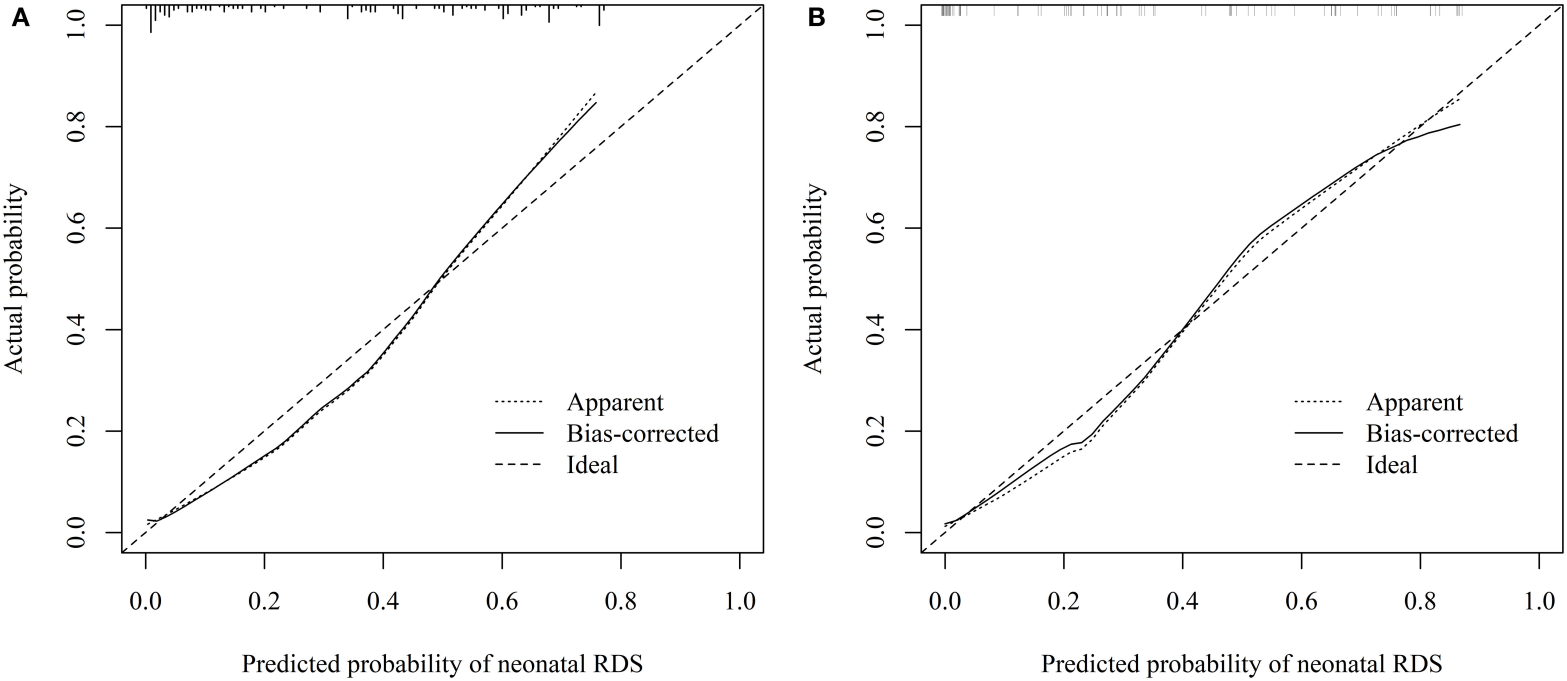
Calibration curves for prediction of LUS. **(A)** training group, **(B)** testing group.

The combined model for predicting the extubation failure among infants with neonatal RDS was carried out according to the predictive factors in the training group, i.e., LUS, gestational age, body length, and birth weight. The AUC of this combined model was 0.871 (95% CI: 0.819–0.922) with a sensitivity of 86.67% (95% CI: 77.80–93.40) and a specificity of 74.31% (95% CI: 66.40–81.20). The AUC of LUS was 0.858 (95% CI: 0.804–0.911) with a sensitivity of 84.00% (95% CI: 73.70–91.40) and a specificity of 80.69% (95% CI: 73.30–86.80). There was no statistical difference in the predictive power between the combined model and LUS (*Z* = 0.880, *P* = 0.379) (Table 5 and Figure 3A). The cutoff value of LUS was 18, suggesting LUS > 18 may be associated with the extubation failure in neonatal RDS. The AUC of LUS for predicting the extubation failure was superior to the AUCs of gestational age, body length, and birth weight. The cutoff values of gestational age, body length, and birth weight were 29 weeks, 37 cm, and 1.19 kg, respectively. The calibration curve of the predictive effectiveness of LUS is shown in Figure 4A based on the training group. The internal validation using the testing set was carried out as shown in Table 5 and Figure 3B. The AUC of LUS was 0.855 (95% CI: 0.817–0.954). It was indicated that LUS had the predictive ability for extubation failure from MV among newborns with RDS. The fitting effect of the calibration curve is listed in Figure 4B, which suggests that the predictive effectiveness of LUS was good.

**Table 5:** The prediction for the extubation failure in RDS infants.

**Variables**	**AUC**	**Sensitivity (95% CI)**	**Specificity (95% CI)**	**Cut-off**	***Z***	***P***
**Training group**						
Combined model	0.871 (0.819–0.922)	86.67 (76.80–93.40)	74.31 (66.40–81.20)	0.306		
LUS	0.858 (0.804–0.911)	84.00 (73.70–91.40)	80.69 (73.30–86.80)	18	0.880	0.379
Gestational age	0.655 (0.588–0.717)	38.67 (27.60–50.60)	88.89 (82.63–93.50)	29	5.365	<0.001
Body length	0.739 (0.710–0.767)	80.94 (75.20–86.60)	56.82 (55.32–58.25)	37	4.345	<0.001
Birth weigh	0.635 (0.567–0.698)	70.67 (59.02–80.60)	49.66 (41.30–58.10)	1.19	5.732	<0.001
**Internal validation**						
Combined model	0.892 (0.826–0.958)	93.55 (78.60–99.20)	79.37 (67.30–88.50)	0.306		
LUS	0.855 (0.817–0.954)	83.87 (66.30–94.50)	85.48 (74.20–93.10)	18	0.248	0.804
Gestational age	0.648 (0.553–0.744)	72.92 (61.53–84.26)	30.34 (19.20–41.43)	29	4.134	<0.001
Body length	0.412 (0.206–0.617)	64.31 (39.20–89.42)	19.00 (2.36–35.80)	37	4.349	<0.001
Birth weigh	0.652 (0.458–0.847)	67.70 (51.30–84.20)	0.649 (52.56–77.32)	1.19	2.296	0.022

*RDS, respiratory distress syndrome; AUC, area under curve; CI, confidence interval; LUS, lung ultrasound score*.

## Discussion

Neonatal RDS is manifested by respiratory dysfunction resulting from the collapse and sharp reduction of residual gas in the alveolar due to the lack of sufficient active substances on the surface of newborn lungs, and it mainly occurs in premature and low birth weight infants. Imaging examination is a common diagnostic technique among newborns with RDS, including chest X-ray, CT, and lung ultrasound (20). Of these, X-ray is a basic diagnostic method for pulmonary lesions, with simple operation and low examination cost, but it is easy to be affected by the overlapping of internal organs in the chest, resulting in insufficient image clarity and unclear display of tiny lung lesions, which is prone to missed diagnosis and misdiagnosis. Both chest X-ray and CT have relatively large radiation damage, which may have a certain adverse effect on the development of neonates (21, 22). Moreover, the poor cooperation of most families made it impossible to perform real-time dynamic detection and repeated scanning on critically ill infants, which affects the diagnostic results. As a non-invasive, dynamic, and real-time imaging technique, ultrasound has been gradually applied in the diagnosis of neonatal pulmonary diseases, such as neonatal RDS, transient tachypnea of new-born, pneumonia, meconium aspiration syndrome, and pneumothorax (20, 23).

Previous studies reported that LUS has good diagnostic accuracy and specificity in comparison with chest X-ray, especially for the quantitative diagnosis of neonatal RDS (24, 25). To date, LUS-related studies focused on the predictive values of LUS in need for surfactants and the severity of neonatal RDS (15, 26). Nonetheless, few studies assessed the role of LUS in the weaning off from MV among babies with RDS. In the current study, a total of 314 infants who underwent MV support were included, with 208 of the extubation success and 106 of the extubation failure. The purpose was to investigate the predictive power of LUS among RDS neonates who failed extubation from MV. The results suggested that gestational age, body length, birth weight, and LUS were the predictive factors of the extubation failure, and a predictive model was conducted to evaluate the risk of extubation failure on the basis of these variables in newborns with RDS. Then, the comparison of predictive effectiveness between the model and LUS was carried out, and no significant difference was observed. The AUC of LUS was 0.858, with a sensitivity of 84.00%, a specificity of 80.69%, and a cutoff value of 18, which was similar to the results of the internal validation. It was indicated that LUS performed the predictive ability for the extubation failure from MV among RDS newborns. When LUS was over 18, the risk of extubation failure from MV may increase in RDS newborns.

The alteration in acoustic patterns is determined by the dynamic change between air and fluids in the lung parenchyma, which is easy to measure (27). LUS can be applied to detect vertical hyperechoic comet-tail B-lines artifacts (BLA) (28), and BLA is commonly considered to be associated with lung aeration and extravascular lung water (EVLW) (29). The change of pulmonary condition can be evaluated by the number of B-lines because B-lines increase with loss of lung aeration and increased EVLW (30). According to Anile et al. (31), the presence of >3 positive lung quadrants was considered a good index to identify EVLW. In Brat et al. (32), the LUS was closely related to the oxygenation status of preterm newborns, and it may be highly reliable to predict the administration of surfactants in preterm infants. What's more, Pang et al. (17) demonstrated that LUS and consolidation areas can be used to grade neonatal RDS and to discriminate neonatal RDS from non-neonatal RDS and can also predict the outcome of applying MV. Tenza-Lozano et al. (33) also mentioned that the feasibility of MV weaning could be assessed by LUS, similar to our findings. It was indicated that LUS is a good predictive tool for the weaning off from MV, and it may be useful for clinicians to intervene early to avoid the extubation failure from MV.

In the present study, we assessed the predictive value of LUS in the extubation failure among RDS infants, which was rarely explored. We discovered that the effectiveness of LUS was similar to that of the combined model in predicting the extubation failure from MV. Given that the operation of LUS was not complicated, the interpretation of LUS diagnosis results has high interobserver agreement even among interpreters with varying levels of experience (34), indicating that LUS may be an effective prediction tool for the extubation outcomes after MV. In addition, some limitations should be warranted caution for interpreting the findings. First, a retrospective cohort study with an internal validation was conducted to assess the effectiveness of LUS in predicting extubation failure among newborns with RDS. Second, LUS is a semiquantitative measurement. The reproducibility of B-line between transducers and between raters was of concern, which depended on the transducer used and interpretation of the raters (35, 36). Third, two pediatricians stored the pulmonary ultrasound images, and the consistency between operators was not evaluated. Further studies with multiple centers, large samples, and perspective designs are needed to explore.

In this study, we found gestational age, body length, birth weight, and LUS were the predictive factors of the extubation failure from MV. The AUC of LUS was 0.858, with a sensitivity of 84.00%, a specificity of 80.69%, and a cutoff value of 18, indicating LUS performed the predictive ability in the extubation failure from MV among newborns with RDS, which may guide pediatricians to conduct early interventions and treatments.

## Data Availability Statement

The raw data supporting the conclusions of this article will be made available by the authors, without undue reservation.

## Ethics Statement

The studies involving human participants were reviewed and approved by the Institutional Review Board (IRB) of Guangdong Second Provincial General Hospital. Written informed consent to participate in this study was provided by the participants' legal guardian/next of kin.

## Author Contributions

ZL, CY, and QM designed the study. ZL wrote the manuscript. QM, BW, XL, and QW collected, analyzed, and interpreted the data. CY critically reviewed, edited, and approved the manuscript. All authors read and approved the final manuscript.

## Funding

This study was supported by the Medical Research Fund of Guangdong Province (A2020064). The funding body did not play a role in designing the study, or collecting, analyzing, or interpreting data, or writing the manuscript.

## Conflict of Interest

The authors declare that the research was conducted in the absence of any commercial or financial relationships that could be construed as a potential conflict of interest.

## Publisher's Note

All claims expressed in this article are solely those of the authors and do not necessarily represent those of their affiliated organizations, or those of the publisher, the editors and the reviewers. Any product that may be evaluated in this article, or claim that may be made by its manufacturer, is not guaranteed or endorsed by the publisher.

## References

[1] Rahtu M Frerichs I Waldmann AD Strodthoff C Becher T Bayford R. Early recognition of pneumothorax in neonatal respiratory distress syndrome with electrical impedance tomography. Am J Respir Crit Care Med. (2019) 200:1060–1. 10.1164/rccm.201810-1999IM 31091957

[2] Sweet LR Keech C Klein NP Marshall HS Tagbo BN Quine D. Respiratory distress in the neonate: Case definition & guidelines for data collection, analysis, and presentation of maternal immunization safety data. Vaccine. (2017) 35:6506–17. 10.1016/j.vaccine.2017.01.046 29150056 PMC5710987

[3] Shao XM Ye HM Qiu XS. Practice of Neonatology (5th edition). Beijing: People's Medical Publishing House (China) (2012).

[4] Stoll BJ Hansen NI Bell EF Shankaran S Laptook AR Walsh MC. Neonatal outcomes of extremely preterm infants from the NICHD Neonatal Research Network. Pediatrics. (2010) 126:443–56. 10.1542/peds.2009-2959 20732945 PMC2982806

[5] Parkash A Haider N Khoso ZA Shaikh AS. Frequency, causes and outcome of neonates with respiratory distress admitted to Neonatal Intensive Care Unit, National Institute of Child Health, Karachi. J Pak Med Assoc. (2015) 65:771–5. 26160089

[6] Smallwood CD Davis MD. Year in review 2018: pediatric mechanical ventilation. Respir Care. (2019) 64:855–63. 10.4187/respcare.07029 31243160

[7] Ding F Zhang J Zhang W Zhao Q Cheng Z Wang Y. Clinical study of different modes of non-invasive ventilation treatment in preterm infants with respiratory distress syndrome after extubation. Front Pediatr. (2020) 25:63. 10.3389/fped.2020.00063 32161744 PMC7053424

[8] Abdel Rahman DA Saber S El-Maghraby A. Diaphragm and lung ultrasound indices in prediction of outcome of weaning from mechanical ventilation in Pediatric Intensive Care Unit. Indian J Pediatr. (2020) 87:413–20. 10.1007/s12098-019-03177-y 32036590 PMC7223651

[9] Xue Y Zhang Z Sheng CQ Li YM Jia FY. The predictive value of diaphragm ultrasound for weaning outcomes in critically ill children. BMC Pulm Med. (2019) 19:270. 10.1186/s12890-019-1034-0 31888586 PMC6937936

[10] Glenski JA Crawford M Rehder K. High-frequency, small-volume ventilation during thoracic surgery. Anesthesiology. (1986) 64:211–4. 10.1097/00000542-198602000-00014 3511770

[11] Ko DR Beom J Lee HS You JS Chung HS Chung SP. Benefits of high-flow nasal cannula therapy for acute pulmonary edema in patients with heart failure in the emergency department: a prospective multi-center randomized controlled trial. J Clin Med. (2020) 9:1937. 10.3390/jcm9061937 32575829 PMC7355695

[12] Fadila M Rajasurya V Regunath H. Ventilator Weaning. Treasure Island, FL: StatPearls Publishing Copyright © 2020 (2020). 28613464

[13] Szymański P Kruczek P Hozejowski R Wais P. Modified lung ultrasound score predicts ventilation requirements in neonatal respiratory distress syndrome. BMC Pediatr. (2021) 21:17. 10.1186/s12887-020-02485-z 33407270 PMC7785923

[14] Sawires HK Abdel Ghany EA Hussein NF Seif HM. Use of lung ultrasound in detection of complications of respiratory distress syndrome. Ultrasound Med Biol. (2015) 41:2319–25. 10.1016/j.ultrasmedbio.2015.04.024 26027895

[15] De Martino L Yousef N Ben-Ammar R Raimondi F Shankar-Aguilera S De Luca D. Lung ultrasound score predicts surfactant need in extremely preterm neonates. Pediatrics. (2018) 142:463. 10.1542/peds.2018-0463 30108142

[16] Vento G Ventura ML Pastorino R Kaam A Carnielli V Cools F. Lung recruitment before surfactant administration in extremely preterm neonates with respiratory distress syndrome (IN-REC-SUR-E): a randomised, unblinded, controlled trial. Lancet Respir Med. (2020) 9:159–66. 10.1016/S2213-2600(20)30179-X 32687801

[17] Pang H Zhang B Shi J Zang J Qiu L. Diagnostic value of lung ultrasound in evaluating the severity of neonatal respiratory distress syndrome. Eur J Radiol. (2019) 116:186–91. 10.1016/j.ejrad.2019.05.004 31153563

[18] Rouby JJ Arbelot C Gao Y Zhang M Lv J An Y. Training for lung ultrasound score measurement in critically ill patients. Am J Respir Crit Care Med. (2018) 198:398–401. 10.1164/rccm.201802-0227LE 29557671 PMC7205011

[19] Moschietto S Doyen D Grech L Dellamonica J Hyvernat H Bernardin GJC. Transthoracic echocardiography with Doppler tissue imaging predicts weaning failure from mechanical ventilation: evolution of the left ventricle relaxation rate during a spontaneous breathing trial is the key factor in weaning outcome. Crit Care. (2012) 16:R81. 10.1186/cc11339 22583512 PMC3580624

[20] Gregorio-Hernandez R Arriaga-Redondo M Perez-Perez A Ramos-Navarro C Sanchez-Luna M. Lung ultrasound in preterm infants with respiratory distress: experience in a neonatal intensive care unit. Eur J Pediatr. (2020) 179:81–9. 10.1007/s00431-019-03470-0 31655870

[21] Ait-Ali L Andreassi MG Foffa I Spadoni I Vano E Picano E. Cumulative patient effective dose and acute radiation-induced chromosomal DNA damage in children with congenital heart disease. Heart. (2010) 96:269–74. 10.1136/hrt.2008.160309 19687017

[22] Kleinerman RA. Cancer risks following diagnostic and therapeutic radiation exposure in children. Pediatr Radiol. (2006) 36(Suppl. 2):121–5. 10.1007/s00247-006-0191-5 16862418 PMC2663653

[23] Liu J Liu F Liu Y Wang HW Feng ZC. Lung ultrasonography for the diagnosis of severe neonatal pneumonia. Chest. (2014) 146:383–8. 10.1378/chest.13-2852 24833216

[24] Smolarova S Kocvarova L Matasova K Zibolen M Calkovska A. Impact of updated European Consensus Guidelines on the management of neonatal respiratory distress syndrome on clinical outcome of preterm infants. Adv Exp Med Biol. (2015) 835:61–6. 10.1007/5584_2014_39 25310949

[25] Barillari A Fioretti M. Lung ultrasound: a new tool for the emergency physician. Intern Emerg Med. (2010) 5:335–40. 10.1007/s11739-010-0381-x 20443081

[26] Vardar G Karadag N Karatekin G. The role of lung ultrasound as an early diagnostic tool for need of surfactant therapy in preterm infants with respiratory distress syndrome. Am J Perinatol. (2020). 38, 1547–1556. 10.1055/s-0040-1714207 32674204

[27] Chen SW Zhang MY Liu J. Application of lung ultrasonography in the diagnosis of childhood lung diseases. Chin Med J. (2015) 128:2672–8. 10.4103/0366-6999.166035 26415808 PMC4736855

[28] Volpicelli G Elbarbary M Blaivas M Lichtenstein DA Mathis G Kirkpatrick AW. International evidence-based recommendations for point-of-care lung ultrasound. Intensive Care Med. (2012) 38:577–91. 10.1007/s00134-012-2513-4 22392031

[29] Lichtenstein DA. Ultrasound in the management of thoracic disease. Crit Care Med. (2007) 35:S250–61. 10.1097/01.CCM.0000260674.60761.85 17446785

[30] Bouhemad B Brisson H Le-Guen M Arbelot C Lu Q Rouby JJ. Bedside ultrasound assessment of positive end-expiratory pressure-induced lung recruitment. Am J Respir Crit Care Med. (2011) 183:341–7. 10.1164/rccm.201003-0369OC 20851923

[31] Anile A Russo J Castiglione G Volpicelli G. A simplified lung ultrasound approach to detect increased extravascular lung water in critically ill patients. Crit Ultrasound J. (2017) 9:13. 10.1186/s13089-017-0068-x 28612302 PMC5469722

[32] Brat R Yousef N Klifa R Reynaud S Shankar Aguilera S De Luca D. Lung ultrasonography score to evaluate oxygenation and surfactant need in neonates treated with continuous positive airway pressure. JAMA Pediatr. (2015) 169:e151797. 10.1001/jamapediatrics.2015.1797 26237465

[33] Tenza-Lozano E Llamas-Alvarez A Jaimez-Navarro E Fernández-Sánchez J. Lung and diaphragm ultrasound as predictors of success in weaning from mechanical ventilation. Crit Ultrasound J. (2018) 10:12. 10.1186/s13089-018-0094-3 29911284 PMC6004341

[34] Brusa G Savoia M Vergine M Bon A Copetti R Cattarossi L. Neonatal lung sonography: interobserver agreement between physician interpreters with varying levels of experience. J Ultrasound Med. (2015) 34:1549–54. 10.7863/ultra.15.14.08016 26254148

[35] Corradi F Via G Forfori F Brusasco C Tavazzi G. Lung ultrasound and B-lines quantification inaccuracy: B sure to have the right solution. Intensive Care Med. (2020) 46:1081–3. 10.1007/s00134-020-06005-6 32189008 PMC7087507

[36] Haaksma ME Smit JM Heldeweg MLA Pisani L Elbers P Tuinman PR. Lung ultrasound and B-lines: B careful! *Intensive Care Med*. (2020) 46:544–5. 10.1007/s00134-019-05911-8 31996959

